# 515 Deficiencies of Rule-based Technology generated Antibiograms and Application in Patients with Prolonged Lengths of Stay

**DOI:** 10.1093/jbcr/irac012.146

**Published:** 2022-03-23

**Authors:** David M Hill, Faisal Arif, Ibrahim Sultan-Ali, Sai R Velamuri

**Affiliations:** Firefighters' Burn Center, Memphis, Tennessee; Firefighter's Burn Center, Memphis, Tennessee; Firefighter's Burn Center, Memphis, Tennessee; Firefighter's Burn Center, Memphis, Tennessee

## Abstract

**Introduction:**

Antibiograms are unit specific annual reports of cumulative pathogen incidence and antibiotic susceptibilities, used to guide selection of empiric antibiotic therapies. Rule-based technology (RBT) expedites data compilation, but follows “first pathogen, per patient” and limits applicability, especially for prescribing antibiotics in units with higher lengths of stay. The objective was to compare the pathogens and susceptibilities of the current automated RBT antibiogram with one manually collected through chart review with additional rules applied.

**Methods:**

This is a single-center, retrospective cohort study utilizing chart review to assess patients admitted to the Burn Center between January 2018 and December 2019 from whom significant bacterial cultures were obtained. Demographics and burn injury characteristics were collected. Treatment data related to the infection and antibiotic usage was also collected. Specific to the culture, timing, site, pathogen, and sensitivity were collected. All cultures within the first 30 days of admission were included. The current RBT antibiogram served as the control. And new antibiogram versions were created using additional rules and compared to the RBT antibiogram.

**Results:**

During the 2 years, 657 patients were admitted. Two-hundred four patients remained after applicable exclusions. Sixty-one percent were excluded due to lack of cultures. Mean age was 50.6 ± 16.5 years and 66% were male. Forty-nine percent were Caucasian. Seventy-two percent were admitted for an acute burn injury with flame as the primary mechanism and a median percent total body surface area of 10 (3, 21). Fifty-nine percent had at least one hospital acquired risk factor with over one-third having recent illicit drug use and one-third having a recent hospitalization. Of the 410 cultures included, 57% were Gram-negative and half were from wound infections. Sensitivities were significantly different when comparing the RBT to those created from significant cultures within 7 days of admission, cultures within 7 days of admission and without hospital-acquired infection risk factors (Figure 1), and those being treated with an initial or subsequent course of antibiotics. Recommended empiric antibiotic changed from double coverage to a single β-lactam with > 90% susceptibility. The susceptibilities between first and subsequent courses were dramatically different (Figure 2)

**Conclusions:**

The antibiogram was significantly different from the RBT version after including factors, such as days since admission, presence of hospital acquired risk factors, or previous antibiotic courses. Before developing an antibiogram or interpreting the output, it is important to consider which automated criteria are utilized, especially for units with extended lengths of stay.

